# Distinct pattern of lymphoid neoplasms characterizations according to the WHO classification (2016) and prevalence of associated Epstein–Barr virus infection in Nigeria population

**DOI:** 10.1186/s13027-021-00378-z

**Published:** 2021-05-24

**Authors:** Ijeoma C. Uzoma, Idowu A. Taiwo, Massimo Granai, Gioia Di Stefano, Ester Sorrentino, Sussana Mannucci, Muheez A. Durosinmi, Stefano Lazzi, Lorenzo Leoncini, Oluyemi Akinloye

**Affiliations:** 1 Molecular-Haematology Laboratory, Department of Medical Laboratory Science, College of Medicine, College of Medicine, University of Nigeria Nsukka, Enugu Campus, Enugu, Nigeria; 2 Genetics Laboratory, Department of Cell Biology and Genetics, Faculty of Science, University of Lagos, Lagos, Nigeria; 3 Centre for Genomics of Non-communicable Diseases and Personalized Healthcare (CGNPH), University of Lagos, Lagos, Nigeria; 4 Institute of Pathology, University Hospital of Tübingen, Tübingen, Germany; 5 Section of Pathology, Department of Medical Biotechnology, University of Siena, Siena, Italy; 6 Department of Haematology, Obafemi Awolowo University Teaching Hospital Complex, Ile-Ife, Nigeria; 7 Clinical Chemistry and Molecular Diagnostics Laboratory, Department of Medical Laboratory Sciences, Faculty of Basic Medical Centre, College of Medicine, University of Lagos, Lagos, Nigeria

**Keywords:** Lymphoid neoplasms, WHO classification, Epstein-Barr virus, Immunohistochemistry, In-situ hybridisation

## Abstract

**Background:**

The present study aimed to classify lymphoid neoplasms according to the latest World Health Organization (WHO) classification and outlining the distribution in Nigeria of different entities. Additionally, the study describes the prevalence of lymphoid neoplasms associated with Epstein-Barr virus (EBV) infection in the Nigerian population.

**Methods:**

We collected 152 formalin-fixed paraffin-embedded (FFPE) tissues diagnosed as lymphoma from 2008 to 2018, coming from three different institutions located within three geopolitical zone in Nigeria. These institutions included the University College Hospital (UCH), Ibadan, Oyo State, the Enugu State University of Science and Technology Teaching Hospital (ESUTH), Enugu, Enugu State, and the Meena Histopathology and Cytology Laboratory (MHCL), Jos, Plateau State.

**Results:**

From the total 152 cases retrieved, 50 were excluded due to insufficient tissue materials or inconclusive antigen reactivity. We confirmed 66 (64.7%) cases as lymphomas out of the remaining 102 FFPE with a male to female ratio of 2:1 and a mean age of 44.4 years. Ten entities were identified, and of these, chronic lymphocytic leukemia (CLL) was the most prevalent category (34.8%). For the diffuse large B-cell lymphomas not otherwise specified (DLBCL, NOS), the germinal centre B–cell type was the most common (71.4%). Ten lymphoma cases (15.2%) were positive for Epstein-Barr virus (EBV), most of which were Hodgkin lymphoma (HL). CLL was common in the Hausa ethnic group, HL in the Yoruba ethnic group, while the Igbo ethnic group had an equal distribution of CLL, HL, and DLBCL diagnosis.

**Conclusion:**

Although the distribution of lymphomas in Nigeria shares some similarities with those of other countries, we described distinct features of some subtypes of lymphomas. Also, the study underscores the need for a more precise diagnosis and classification of lymphoid neoplasms in Nigeria using the latest WHO classification.

**Supplementary Information:**

The online version contains supplementary material available at 10.1186/s13027-021-00378-z.

## Introduction

Lymphomas are a heterogeneous group of neoplasms based on genetically complex mechanisms that can be generally categorized as Hodgkin lymphomas (HL) or non-Hodgkin lymphomas (NHL) [1]. Also, lymphomas show a broad spectrum of clinical and pathological presentations, and in sub-Saharan Africa, they continue to be one of the primary causes of morbidity and mortality [2, 3]. Precisely, Nigeria has recorded an increasing incidence of NHL. NHL is reported as the 5th most common cancer by the national cancer registries [4, 5], and their definition and characteristics are ever-increasing and more complex since molecular and genetic features are constantly being identified to allow a more accurate diagnosis and prognosis [6].

Lymphoid neoplasms show ethnic and geographical differences in their distribution and molecular characteristics [7]. Unlike developing countries, substantial efforts have been made in developed nations to match each lymphoma category with advanced diagnostic tools: diagnosis, therapy, and prognosis [8, 9].

The classifications of Tumors of Haematopoietic and Lymphoid Tissues are now based on the latest investigations to identify and describe the tumor characteristics and were previously captured in the Revised European-American Classification of Lymphoid Neoplasms (REAL) system of classification in 1994 that was subsequently integrated into the World Health Organization classification (WHO) [6, 10–12].

Some infectious agents have been implicated as drivers of lymphomagenesis, including the Epstein Barr virus (EBV), human herpesviruses 8 (HHV-8), human immunodeficiency virus (HIV), and *Plasmodium falciparum* [13, 14]. EBV is the most common etiological viral agent associated with lymphomagenesis and is present in endemic and sporadic Burkitt lymphoma (BL), Hodgkin lymphoma (HL), and diffuse large B-cell lymphoma, not otherwise specified (DLBCL, NOS) [15]. The EBV Encoded RNA (EBER) is present at the latent stage of developing the virus in EBV infected individuals [16]. The detection of all of these pathogens enables appropriate therapeutic measures to reduce disease burden and improve the overall survival of lymphoma patients in Nigeria. There is a need even more to understand the distribution and spread of these malignancies in Nigeria using more thorough investigations. The lack or limited availability of these tools in low and middle-income countries makes accurate diagnosis and classification of lymphoid neoplasms difficult or practically impossible. Consequently, in these countries, patients’ overall survival with lymphoid malignancies is meager [17, 18]. Many Nigerian institutions still employ only histo-morphological characterization, leading to misdiagnosis and resulting in improper treatment [19].

The present study aimed to reclassify lymphoma cases from three different medical institutions in Nigeria that were diagnosed by analyzing only the morphological features with Hematoxylin-Eosin (H&E) staining and then evaluate the differences between the original and the revised diagnosis. Moreover, we aimed to determine in the study cohort the prevalence of EBV in different lymphoma subtypes and the frequency of DLBCL subtypes using the Hans algorithm [20].

## Material and methods

### Study cohort

Formalin-fixed paraffin-embedded (FFPE) biopsies, including their demographic data, were collected from tertiary facilities of three different geopolitical areas in Nigeria: University College Hospital, Ibadan, Oyo State, Enugu State University of Science and Technology Teaching Hospital (ESUTH), and Meena Histopathology and Cytology Laboratory, Jos, Plateau State. Ethical committee approvals were obtained from the University of Nigeria Teaching Hospital, Enugu (UNTH-NHREC/05/01/2008B), Ministry of Health, Ibadan, Oyo State (AD13/479/1138), and Meena Histopathology and Cytology Laboratory, Jos, Plateau State (MEENALAB/AEC/177). A total of 152 FFPE morphologically diagnosed as lymphoid neoplasms in the already listed institutions between 2008 and 2018 were obtained. The revised classification was made according to the latest WHO classification of Tumors of Lymphoid Tissues. The statistical analysis was done with IBM, SPSS version 25.0 software package (IBM® Software). The level of statistical significance was set at *p* < 0.05. Inferential statistics were t-test in case of 2 groups and ANOVA in case of 3 or more groups with continuous normally distributed variables. Significant ANOVA results were followed by Tukey post hoc test for paired comparison. Association between variables was by Pearson product-moment correlation (r) and Chi-square (X^2^) test for independence.

### Histology and immunohistochemistry (IHC)

4 μm sections were cut and used for H&E staining and immunohistochemistry (IHC) from each block. All slides were reviewed by two expert hematopathologists (LL, SL). Cases with insufficient or not optimal material were excluded from the study. The immunohistochemical analysis was performed with a panel of 25 antibodies commonly used in NHL and HL cases, using the Ventana staining system (Ventana, Tucson, AZ, USA).

### Detection of Epstein - Barr virus (EBV)

According to the manufacturer’s instruction, EBV was detected by in-situ-hybridization (ISH) using the INFORM EBER probe (Ventana Medical Systems, Inc., Tucson, AZ, USA). The slides were stained using an automated stainer (Benchmark Ultra, Ventana Medical Systems, Inc., Tucson, AZ, USA). The visualization was by ISH iView Blue Detection kit (Ventana Medical Systems, Inc., Tucson, AZ, USA) with alkaline phosphatase 5-bromo-4-chloro-3-indolyl phosphate/nitro blue tetrazolium (BCIP NBT/) substrate. Nuclear Fast red (Ventana Medical Systems, Inc., Tucson, AZ, USA) was used as a contrast. Appropriate positive and negative controls were set up parallel to the section analyzed.

## Results

### Study cohort and clinical data

**Fig. 1:**
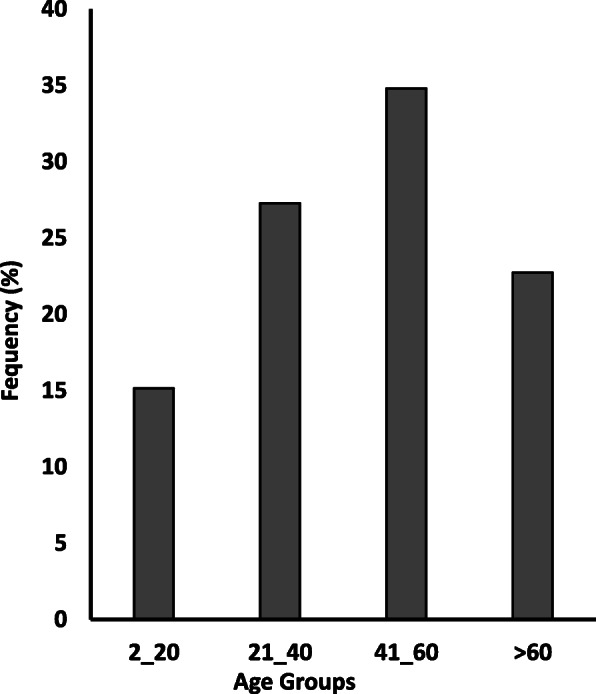
Occurrence of lymphoid neoplasm and age distribution

A total of 152 cases were analyzed. From the study cohort’s total number, 50 cases were excluded due to insufficient tissue materials or inadequate antigen reactivity. Of the remaining 102 samples, after using IHC, in only 66 cases, a lymphoma diagnosis was established. The remaining 36 cases were confirmed with the following diagnosis: 22 reactive lymphadenopathies, 2 Castleman disease (CD), 2 Rosai-Dorfman disease (RDD), and 10 cases as non-lymphoid malignancies. Among the 66 lymphoma cases, 44 (66.7%) and 22 (33.3%) were male and female, respectively, with a male to female ratio of 2:1 (Table 1). All the lymphoma subtypes showed a male predominance with higher ratios in HL (3.3:1) and BL (4:1). The lymphoma cases showed an age range between 2 and 85 years with a mean age of 44.4 ± 20.1 years that was not significantly different (*p* = 0.295) among the two genders (male: 42.6 years; female: 48.1 years). As shown in Fig. 1, most lymphoid neoplasm (34.8%) occurred within the age group of 41–60 years. The mean age of chronic lymphocytic leukemia (CLL) patients (55.7 years) was significantly different from the mean ages of patients with BL (27.6 years; *p* = 0.009) and HL (33.8 years; *p* = 0.001) as opposed to the mean age of patients with DLBCL, NOS (*p* = 0.304).

**Table 1:** .

2016 WHO LYMPHOMA CLASSIFICATION	TOTAL OF CASES	CLL	HL	DLBCL, NOS	BL	SMZL	ENMZL	B-LBL	AITL	FL	LPL
**Number** (%)	66	23 (34.8)	17 (25.8)	14 (21.2)	5 (7.6)	2 (3)	1 (1.5)	1 (1.5)	1 (1.5)	1 (1.5)	1 (1.5)
**Gender** (%)	M = 44 (66.7%) F = 22 (33.3%)	12 (18.18) 11 (16.6)	13 (19.7) 4 (6)	9 (13.6) 5 (7.5)	4 (6) 1 (1.5)	1 (1.5) 1 (1.5)	1 (1.5) 0	1 (1.5) 0	1 (1.5) 0	1 (1.5) 0	1 (1.5) 0
**M: F** (*p* = 0.295)	2:1	1.1:1	3.3:1	1.8:1	4:1	1:1	M	M	M	M	M
**Age** Age range Mean age (*p* = 0.574)	2–85 yrs. 44.1 yrs	36–78 yrs. 55.7 yrs	4–64 yrs. 33.8 yrs	7–85 yrs. 46.1 yrs.	7–65 yrs. 27.6 yrs	N.A.	N.A.	N.A.	N.A.	N.A.	N.A.
**Nodal** (%)	55 (83.3%)	19 (34.5)	17 (30.9)	12 (21.8)	4 (7.3)	0	0	1 (1.8)	1 (1.8)	1 (1.8)	0
**Extranodal** (%)	11 (16.7%)	4 (36.4)	0	2 (18.2)	1 (9.1)	2 (18.2)	1 (9.1)	0	0	0	1 (9.1)
**EBV** (%) (*p* = 0.023)	10 (1.5%)	0	6 (60)	1 (10)	3 (30)	0	0	0	0	0	0

Also, in Table 1, nodal involvement (83.3%) compared to extranodal sites (16.7%) was more frequently observed in the different tumor types. Instead, the extranodal localizations according to the different lymphoid neoplasms were: 4 cases of CLL (omental mass, ovarian mass, breast, and colon), 2 for DLBCL, NOS (jaw and non-nodal tissue mass), 1 for BL (ovary), 2 for splenic marginal zone lymphoma (SMZL) (spleen), 1 for extranodal marginal zone lymphoma (ENMZL) (conjunctiva), and 1 for lymphoplasmacytic lymphoma (LPL) (scalp mass).

### Revised classification with immunohistochemistry of the 66 cases with lymphoma diagnosis

**Fig. 2:**
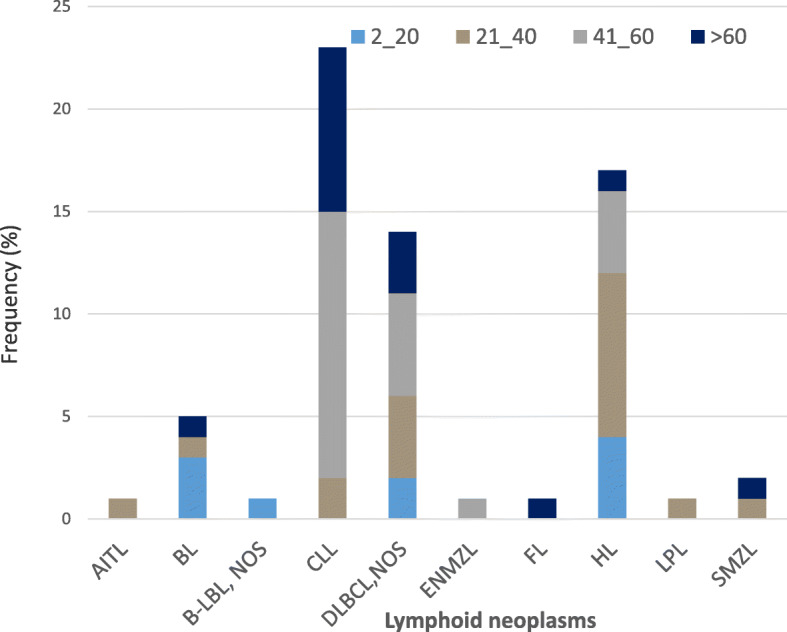
Frequency of Lymphoid neoplasm subtypes within age groups. *CLL/SLL*-Chronic lymphocytic leukemia/small lymphocytic lymphoma, *HL*- Hodgkin lymphoma, *DLBCL, NOS*-Diffuse large B-cell lymphoma, not otherwise specified, *BL*- Burkitt lymphoma, *SMZL*- Splenic marginal zone lymphoma, *ENMZL*- Extra nodal marginal zone lymphoma, *FL*-Follicular lymphoma, *LPL*- Lymphoplasmacytic lymphoma, *AITL*-Angioimmunoblastic T-cell lymphoma, *B-LBL*- B- lymphoblastic lymphoma

The number of cases that were concordant and discordant from the previous diagnosis is shown in Table 2. The list of the antibody that was employed to reclassify are shown in Table 3. A total of 65 (98.5%) cases originated by B lymphocytes while only one by T lymphocytes. The majority of the reclassified lymphomas were CLL (*n* = 23; 34.8%), and a greater proportion (65.2%) of these cases came from the Hausa ethnic group (Table 4) involving mostly nodal sites (34.5%). Only one case of the 23 CLL cases presented a concordant diagnosis while the other 22 (95.6%) presented a discordant diagnosis (Supplementary Table 1). 17 cases of HL/HD (25.8%) were identified with 5 (29.4%) cases that presented a concordant diagnosis and the remaining 12 (70.6%) cases of HL/HD were actually discordant from the original diagnosis (Supplementary Table 2). The highest incidence of HL/HD occurred within the age group of 21–40 years (Fig. 2), with a major frequency in the Yoruba ethnic group (52.9%) as shown in Table 4. The distribution of the HL subtypes was: 4 (23.5%) nodular lymphocyte-predominant Hodgkin lymphoma (NLPHL), 1 (5.9%) lymphocyte rich classical Hodgkin lymphoma (LRCHL), 10 (58.8%) of mixed cellularity classical Hodgkin lymphoma (MCCHL) and 2 (11.8%) nodular sclerosis classical Hodgkin lymphoma (NSCHL). EBV infection was noticed in 6 cases of MCCHL. Following IHC and expert revision, 10 cases (71%) out of 14 of the DLBCL group presented a discordant diagnosis (Supplementary Table 3). Instead, all the BL cases showed a concordant diagnosis in 2 cases (40%) and the rest were discordant (*n* = 3; 60%), as shown in Supplementary Table 4. For the Igbo ethnic group, there was an identical distribution in the number of CLL (33.3%), DLBCL, NOS (33.3%), and HL/HD (33.3%) as presented in Table 4.

**Table 2:** Original diagnosis and revised diagnosis after expert revision and immunohistochemistry

ORIGINAL DIAGNOSIS													
	BL ***n*** = 2	CLL ***n*** = 5	AITL ***n*** = 2	DLBCL ***n*** = 12	HL/HD ***n*** = 6	PCM ***n*** = 1	Reactive ***n*** = 15	Carcinoma ***n*** = 1	FL ***n*** = 1	NK lymphoma ***n*** = 1	LPL ***n*** = 1	MALT ***n*** = 2	NHL ***n*** = 53
REVISED DIAGONSIS													
**CLL** ***n*** = **23**	–	1	–	4	–	–	1	1	–	–	–	–	16
**HL** ***n*** **= 17**	–	–	1	1	5	–	2	–	–	–	–	–	8
**DLBCL** ***n*** **= 14**	–	1	–	4	1	–	1	–	–	–	–	–	7
**BL** ***n*** = **5**	2	–	–	1	–	–	–	–	–	–	–	–	2
**SMZL** ***n*** = **2**	–	2	–	–	–	–	–	–	–	–	–	–	–
**ENMZL** ***n*** = ***1***	–	–	–	–	–	–	–	–	–	1	–	–	–
**B-LBL** ***n*** = **1**	–	–	–	–	–	–	1	–	–	–	–	–	–
**AITL** ***n*** = **1**	–	–	–	1	–	–	–	–	–	–	–	–	–
**FL** ***n*** = **1**	–	–	–	–	–	–	–	–	–	–	–	–	1
**LPL** ***n*** = **1**	–	–	–	–	–	1	–	–	–	–	–	–	–
**Sarcoma** ***n*** = **4**	–	–	–	–	–	–	–	–	–	–	–	–	4
**Metastasis** ***n*** = **6**	–	–	1	1	–	–	–	–	–	–	–	1	3
**CD** ***n*** = **2**	–	–	–	–	–	–	–	–	1	–	1	–	–
**RDD** ***n*** = **2**	–	–	–	–	–	–	–	–	–	–	–	–	2
**Reactive** ***n*** **= 22**	–	1	–	–	–	–	10	–	–	–	–	1	10

*AITL* Angioimmunoblastic T-cell lymphoma, *BL* Burkitt lymphoma, *B-LBL* B-lymphoblastic leukaemia/lymphoma, *CD* Castleman disease, *CLL* chronic lymphocytic leukemia, *DLBCL* diffuse large B-cell lymphomas, *ENMZL* extranodal marginal zone lymphoma, *FL* follicular lymphoma, *HL* Hodgkin lymphoma, *HD* Hodgkin disease, *LPL* lymphoplasmacytic lymphoma, *NHL* non-Hodgkin lymphomas, *PCM* plasmacytoma, *RDD* Rosai-Dorfman disease, *SMZL* splenic marginal zone lymphoma

**Table 3:** List of antibodies used

ANTIBODY	COMPANY	CLONE	DILUTION
Bcl-2	Roche Ventana	SP66	ready to use
Bcl-6	Cell Marque	GI191E/A8	1:50
CD 10	Roche Ventana	SP67	ready to use
CD 138	Roche Ventana	B-A38	ready to use
CD 15	Roche Ventana	MMA	ready to use
CD 20	Roche Ventana	L-26	ready to use
CD 21	Roche Ventana	2G9	ready to use
CD 23	Roche Ventana	SP23	ready to use
CD 3	Roche Ventana	2GV6	ready to use
CICLINA D	Roche Ventana	SP4-R	ready to use
C-MYC	Roche Ventana	EP121	ready to use
HHV8	Roche Ventana	13B10	ready to use
KAPPA	Cell Marque	L1C1	1:20
KI-67	Roche Ventana	30 9	ready to use
LAMBDA	Cell Marque	LAMB14	1:50
IGA	Cell Marque	2652	1:50
IGG	Cell Marque	2653	1:50
IGM	Cell Marque	2654	1:50
PAX5	Roche Ventana	SP34	ready to use
PD-1	Cell Marque	NAT105	1:20
S-100	Roche Ventana	4C4.9	ready to use
TDT	Cell Marque	TDT	1:100

**Table 4:** Lymphoid neoplasm distribution in different population in Nigeria

	TOTAL (66)	CLL (23)	HL (17)	DLBCL, NOS (14)	BL (5)	SMZL (2)	ENMZL (1)	B-LBL (1)	AITL (1)	FL (1)	LPL (1)
**Hausa/MHCL** **(*n*** **= 59)**	29 (43.9%)	15 (65.2%)	5 (29.4%)	7 (50%)	2 (40%)	0	0	0	0	0	0
**Igbo/ESUTH** **(*n*** **= 46)**	9 (13.6%)	3 (13%)	3 (17.6%)	3 (21.4%)	0	0	0	0	0	0	0
**Yoruba/UCH** **(*n*** **= 47)**	28 (42.4%)	5 (21.7%)	9 (52.4%)	4 (28.6%)	3 (60%)	2 (100%)	1 (100%)	1 (100%)	1 (100%)	1 (100%)	1 (100%)

**Fig. 3:**
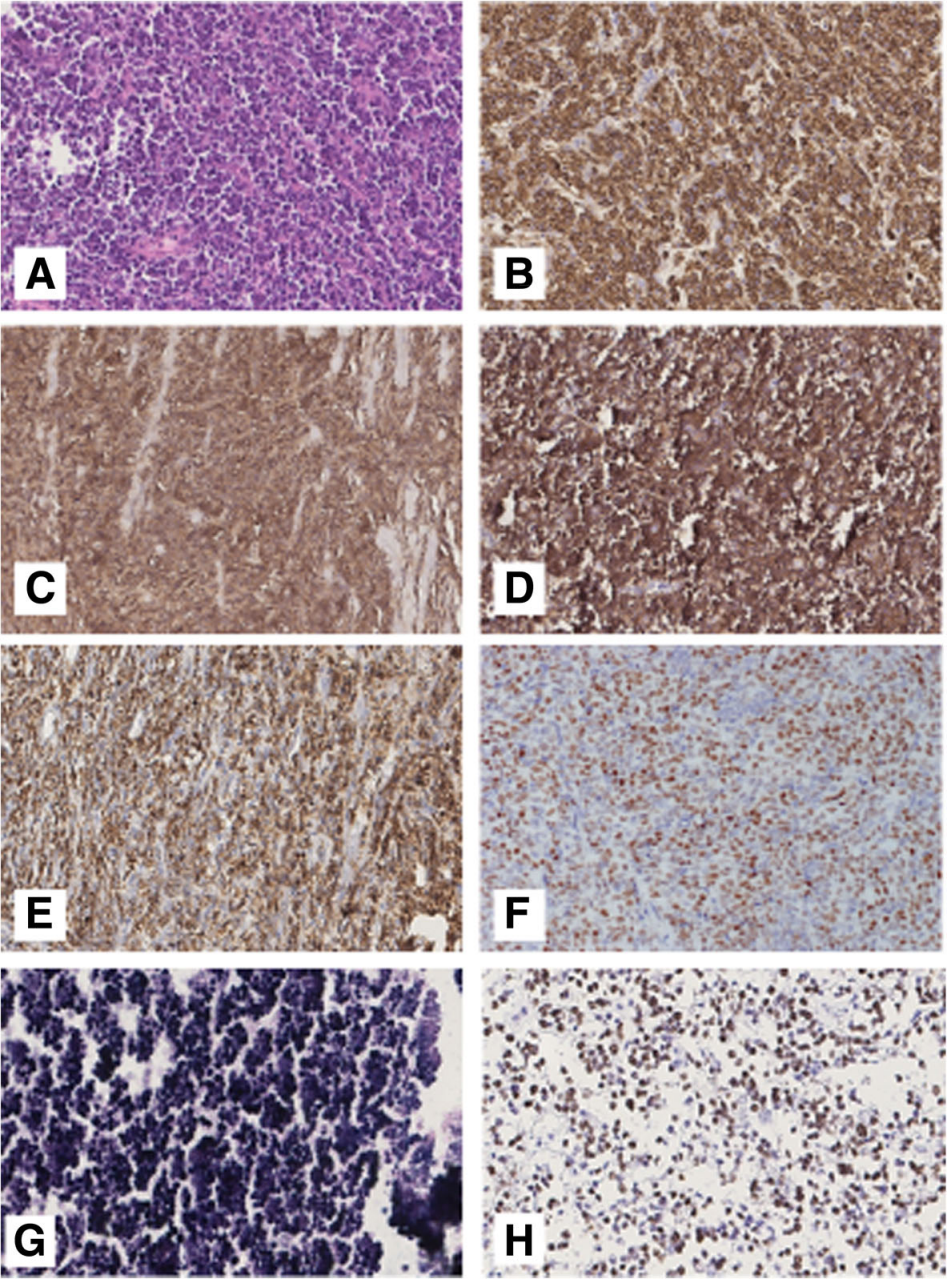
Example of Diffuse Large B-cell Lymphoma. **a**. HE routine stain (× 20). The neoplastic cell express CD20,CD10, BCL6, BCL2, MYC, EBER with high proliferation index evaluated with Ki67 (respectively in **b**, **c**, **d**, **e**, **f, g**, **h** × 20)

**Fig. 4:**
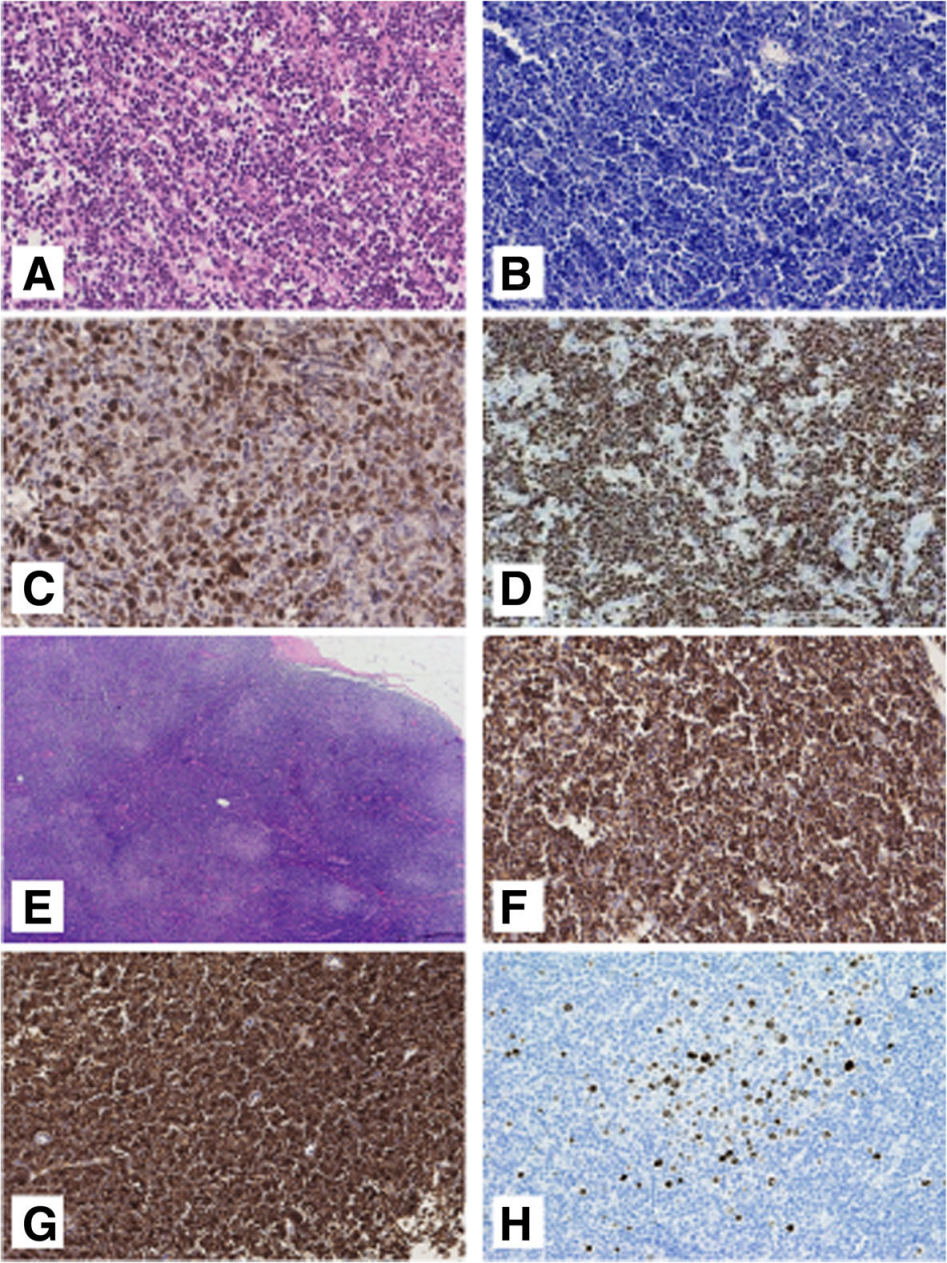
**a** Burkitt lymphoma (HE routine stain × 20). **b** Giemsa stain (× 20). **c** C-MYC expression (× 40). **d**. Ki67 positivity (× 20). **e** Chronic lymphocytic leukemia (HE routine stain × 5). **f** CD5 expression (× 20). **g** CD23 expression (× 20). **h** Cyclin D1 expression in the proliferation centre (× 20)

**Fig. 5:**
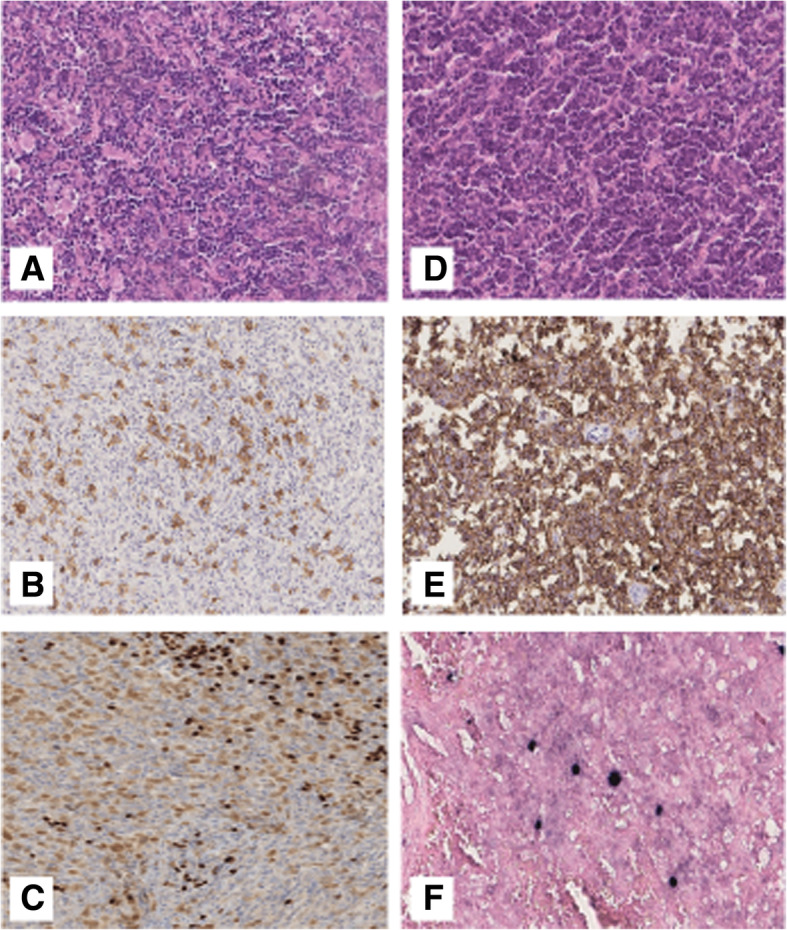
**a** Classical Hodgkin Lymphoma (HE routine stain × 20). **b** The neoplastic cells are positive for CD30 (× 20), **c** Weak positivity for PAX5. **d** Angioimmunoblastic T-cell lymphoma (HE routine stain × 20), **e** CD10 expression (× 20), **f** Scattered large cells positive for EBER (× 40)

The histologic diagnosis of the remaining 6 entities (Supplementary Table 5) were discordant with the final diagnosis according to the latest WHO classification: 2 SMZL (3%), 1 ENMZL (1.5%), 1 (1.5%) follicular lymphoma (FL), 1 LPL (1.5%), 1 (1.5%) B-lymphoblastic leukemia/lymphoma (B-LBL) and 1 (1.5%) Angioimmunoblastic T-cell lymphoma (AITL). The representative slides of the H&E and IHC are shown in Figs. 3, 4 and 5.

### Revised diagnosis of rare conditions and non-lymphoid neoplasm

The non-lymphoid malignancies and other rare conditions that were more accurately diagnosed using IHC are shown in Supplementary Table 6. Apart from the identified rare conditions such as Castleman Disease (*n* = 2; 14.3%), Rosai Dorfman Disease (n = 2; 14.3%), the rest of the cases were metastases of carcinomas (*n* = 6; 43%) and sarcomas (*n* = 4; 28.6%).

### Cell of origin (COO)

The COO determined by the Hans algorithm demonstrated that 10 (71.4%) cases were of the Germinal Center type (GCB), 3 (21.4%) were Non-Germinal Centre (non-GCB) type, and 1 (7.1%) case could not be determined (Supplementary Table 3). Also, in both GCB and non-GCB subtypes, two of the DLBCL, NOS (14.3%), presented co-expression of c-MYC protein and BCL2 (“double expressors”).

### Frequency of Epstein-Barr-virus in the lymphoma subtypes

EBV was observed only in 10 cases (15.2%): 6 HL (60%), 3 BL (30%), and one case of DLBCL, EBV+. All the EBV positive cases of HL were MCCHL. The association between the presence of EBV and lymphoid neoplasm subtypes was statistically significant (*p* = 0.023).

## Discussion

Detailed immunohistochemical characterization is needed to achieve a precise diagnosis to guarantee an appropriate therapy for each lymphoid neoplasm, and this is often lacking in most tertiary hospitals in Nigeria [19]. This subject matter was an inspiration to re-assess all the lymphomas diagnoses that were made using only morphologic features with H&E staining, which is mainly the only investigation available in many institutions in Nigeria.

In the present study, we aimed to reclassify the lymphoma cases adopting the latest WHO classification of lymphoid neoplasms, thus becoming the first study in Nigeria to provide updated data according to the WHO classification of lymphoid neoplasm. The majority of the classified lymphomas occurred within the age range of 41–60 years and were primarily from nodal sites, and these findings are similar to results from other populations [7]. The mean age of 44.4 years obtained in this article is comparable to other studies [21, 22]. A male predominance in the reclassified lymphoma cases and a lower prevalence of lymphoid neoplasms in females have been linked to the influence of estrogen on anti-tumor immune response [7, 23–25].

Interestingly, we observed a higher prevalence of CLL in our investigated population that differed from previous studies’ results, where a higher incidence of DLBCL was reported [21, 26, 27]. The majority of the CLL cases were present in the Hausa group. HL was more frequently observed in the Yoruba group, while there was an identical distribution in the lymphoma subtypes identified in the Igbo group. Evidence from previous studies of stereotyped B cell receptors (BCR) in CLL suggests that common antigenic stimulation could be responsible for the pathogenesis of CLL [28]. It remains unknown whether this antigenic stimulation has ethnic diversity or genetic differences. Therefore, it may be interesting to investigate the frequency of possible common antigenic stimulation in CLL in this ethnic group compared to others.

After CLL, the second most frequent lymphoma was HL, and MCCHL was the most recurrent subtype, that was also commonly associated with EBV infection. Although the HIV status of the studied population was not known, an earlier study had reported a low prevalence of HIV infection among adults in Nigeria [29]. Another study in the Northern part of Nigeria stated a 54.5% frequency of lymphomas correlated to EBV infection [26]. Moreover, the higher prevalence of the MCCHL subtype of HL compared to other HL subtypes was also similar to other populations [27, 30, 31].

Instead, limited information is available on the frequency and distribution of DLBCL, NOS among Nigerian patients. In this study, we assessed the frequency of DLBCL, NOS and these were sub-classified based on the Hans algorithm. The proportion of the DLBCL, NOS GCB type was more recurrent than the non-GCB type. This data is somewhat different from several reports conducted on other sub-Saharan African, Asian, and Western cohorts, where a higher prevalence of the DLBCL, NOS non-GCB type is observed [20, 32–35]. The co-expression of c-MYC and BCL2 proteins (double expressor) was seen in just two cases of the DLBCL, NOS. Even though double expressor DLBCL, NOS is commonly present in the non-GCB subtype, our result did not present any preference for a particular subset since the two double expressers were observed in both subgroups. DLBCL, NOS “double expressor” seems to correlate with a worst prognosis, but the double expression of these two proteins is not necessarily a surrogate indicator of double translocation of *c-MYC* and *BCL2* genes (“double-hit lymphomas”) [36, 37]. Also, the 2 DLBCL “double expressor” cases were identified in nodal sites and this result is similar to earlier studies where a higher frequency of nodal involvement was appreciated [7, 38, 39].

The low frequency of AITL, FL, MZL, and B-LBL, NK-lymphomas in this study are consistent with similar distributions reported in prior African reports [40, 41]. Additionally, the low prevalence of EBV in lymphoma cases is also in line with earlier studies in Nigeria, where a decreased incidence of EBV-correlated lymphoid neoplasms was noticed [21, 26, 42]. Despite being classified as an endemic EBV area, the underlying causes for the EBV-associated lymphoma cases in Nigeria have not been highlighted yet. However, studies have shown that the former ideas of confining EBV infection to Africa and linking it to only BL were oversimplified [43].

The limit of the present study is the fact that the study cohort may not necessarily be representative of the true epidemiology of lymphoid neoplasms in Nigeria since we characterized only available cases rather than doing a prospective assessment. However, the study shows that classification of lymphomas based only on morphological features as it is commonly performed in developing countries, especially in Nigeria, reduces accurate diagnosis, thereby affecting patient management. The diagnostic approach has improved over the years, from the earliest attempt in 1942 by Gall and Mallory to the establishment in 1982 of the Working Formulation (WF) classification system, which in 1994 was later replaced by the Revised European-American (REAL) classification of lymphoid neoplasms [44–46]. The harmonization and the update of the REAL classification by the WHO have provided a more uniform classification for lymphoid neoplasms [6, 11, 47]. The importance of immunohistochemistry in diagnosing lymphoma in Nigeria has been emphasized in several studies carried out in different centers [19, 21, 48, 49].

Furthermore, prior studies have proposed diagnostic algorithms to support minimal immunohistochemical panels to achieve a B-cell lymphoma diagnosis, mainly in institutions with limited resources, before considering consultation in specialized centers [50]. The present study demonstrates how a more collaborative effort is necessary to incorporate routine immunohistochemical analysis to achieve accurate lymphoma diagnosis in Nigeria. Specific diagnosis could be achieved in specialized regional centers of excellence, where advanced tests can be performed, and in this manner, sparse and expensive resources like antibodies can be pulled together to serve a broader population.

## Conclusion

The present study describes morphology-based diagnosis in resource-constrained hospitals in Nigeria that were reclassified according to the WHO after immunohistochemical assessment and also showing different distribution of lymphoid neoplasm in Nigeria. Furthermore, we found interesting distributions and variability of some lymphoid tumors from the previously available literature. In our study cohort, CLL is the most frequent lymphoma, and this data underscores the prevalence of indolent lymphomas rather than aggressive lymphoproliferative neoplasms. Moreover DLBCL, NOS GCB type was the most common, although to evaluate the reliability of this information more investigations with larger cohorts are required. Also, we have documented that the association of EBV with lymphomas in this region is surprisingly low. Implementing the updated WHO classification in developing countries, especially Nigeria, will refine the diagnostic process and improve patient management in lymphoid neoplasms.

## Supplementary Information

**Additional file 1.**

**Additional file 2.**

**Additional file 3.**

**Additional file 4.**

**Additional file 5.**

**Additional file 6.**

## Data Availability

This is not applicable to this study.
