# Supplementary material for: Distinct pattern of lymphoid neoplasms characterizations according to the WHO classification (2016) and prevalence of associated Epstein–Barr virus infection in Nigeria population
Source: Infect Agent Cancer. 2021 May 24;16:36. doi: 10.1186/s13027-021-00378-z (PMC8142647; doi:10.1186/s13027-021-00378-z)
Supplement: Supplementary file 1 — Additional file 1. [file 13027_2021_378_MOESM1_ESM.docx]

**Supplementary Table 1:** Discordant diagnosis for Chronic Lymphocytic Leukemia (n=22)

|  | Age | Gender | Biopsy site | Previous diagnosis | Revised diagnosis |
| --- | --- | --- | --- | --- | --- |
| 1 | 45 | M | Axillary lymph node | NHL | CLL |
| 2 | 63 | M | Cervical Lymph node | Reactive | CLL |
| 3 | 68 | F | Mesenteric lymph node | NHL | CLL |
| 4 | 50 | F | Omental mass | NHL | CLL |
| 5 | 52 | F | Inguinal lymph node | NHL | CLL |
| 6 | 59 | M | Cervical lymph node | NHL | CLL |
| 7 | 52 | M | Cervical lymph node | NHL | CLL |
| 8 | 45 | F | Inguinal lymph node | NHL | CLL |
| 9 | 73 | F | Cervical lymph node | NHL | CLL |
| 10 | 51 | F | Ovarian mass | NHL | CLL |
| 11 | 61 | F | Cervical lymph node | NHL | CLL |
| 12 | 56 | F | Inguinal lymph node | NHL | CLL |
| 13 | 78 | M | Cervical lymph node | NHL | CLL |
| 14 | 70 | M | Cervical lymph node | DLBCL | CLL |
| 15 | 53 | M | Colon | DLBCL | CLL |
| 16 | 38 | F | Breast | Carcinoma | CLL |
| 17 | 52 | M | Cervical lymph node | NHL | CLL |
| 18 | 65 | M | Cervical lymph node | NHL | CLL |
| 19 | 70 | M | Left inguinal lymph node | DLBCL | CLL |
| 20 | 52 | M | Inguinal lymph node | DLBCL | CLL |
| 21 | 36 | F | Cervical lymph node | NHL | CLL |
| 22 | 42 | M | Cervical lymph node | NHL | CLL |
