# Supplementary material for: Distinct pattern of lymphoid neoplasms characterizations according to the WHO classification (2016) and prevalence of associated Epstein–Barr virus infection in Nigeria population
Source: Infect Agent Cancer. 2021 May 24;16:36. doi: 10.1186/s13027-021-00378-z (PMC8142647; doi:10.1186/s13027-021-00378-z)
Supplement: Supplementary file 2 — Additional file 2. [file 13027_2021_378_MOESM2_ESM.docx]

**Supplementary Table 2:** Discordant diagnosis in Hodgkin Lymphoma (n=12)

|  | Age | Gender | Biopsy site | Previous diagnosis | Revised diagnosis |
| --- | --- | --- | --- | --- | --- |
| 1 | 60 | F | Cervical lymph node | NHL | HL – Nodular Sclerosis |
| 2 | 60 | M | Axillary lymph node | DLBCL | HL – Mixed cellularity |
| 3 | 22 | M | Cervical lymph node | NHL | HL – Mixed cellularity |
| 4 | 48 | M | Cervical lymph node | NHL | HL – Mixed cellularity |
| 5 | 9 | M | Cervical lymph node | NHL | Nodular Lymphocyte Predominant |
| 6 | 53 | M | Bilateral cervical lymph node | Angioimmunoblastic lymphoma | Nodular Lymphocyte Predominant |
| 7 | 39 | M | Lymph node | NHL | HL – Nodular Sclerosis |
| 8 | 40 | M | Lymph node | NHL | HL – Mixed cellularity |
| 9 | 64 | F | Lymph node | Reactive | HL – Mixed cellularity |
| 10 | 17 | F | Lymph node | NHL | HL – Lymphocyte rich |
| 11 | 4 | M | Lymph node | Reactive | HL – Mixed cellularity |
| 12 | 39 | M | Lymph node | NHL | HL – Mixed cellularity |
