# Supplementary material for: Distinct pattern of lymphoid neoplasms characterizations according to the WHO classification (2016) and prevalence of associated Epstein–Barr virus infection in Nigeria population
Source: Infect Agent Cancer. 2021 May 24;16:36. doi: 10.1186/s13027-021-00378-z (PMC8142647; doi:10.1186/s13027-021-00378-z)
Supplement: Supplementary file 3 — Additional file 3. [file 13027_2021_378_MOESM3_ESM.docx]

**Supplementary Table 3:** Discordant diagnosis of Diffuse Large B-cell Lymphoma (n=10)

|  | Age | Gender | Biopsy site | Previous diagnosis | Revised diagnosis |
| --- | --- | --- | --- | --- | --- |
| 1 | 51 | F | Cervical lymph node | NHL | DLBCL, NOS |
| 2 | 43 | F | Cervical lymph node | N.A.^1^ | DLBCL, NOS (GCB type) |
| 3 | 59 | M | Cervical lymph node | NHL | DLBCL, NOS (GCB type) |
| 4 | 7 | F | Soft tissue | NHL | DLBCL, NOS (GCB type) |
| 5 | 50 | M | Axillary lymph node | NHL | DLBCL, NOS (GCB type) |
| 6 | 50 | M | Cervical lymph node | NHL | DLBCL, NOS (GCB type) |
| 7 | 13 | M | Right cervical lymph node | NHL | DLBCL, NOS (non-GCB type) |
| 8 | 62 | M | Axillary lymph node | HL | DLBCL, NOS (GCB type) |
| 9 | 39 | M | Cervical lymph node | Reactive | DLBCL, NOS (GCB type) |
| 10 | 37 | F | Cervical lymph node | NHL | DLBCL, NOS (non-GCB type) |

**^1^** N.A.= not available
