# Supplementary material for: Distinct pattern of lymphoid neoplasms characterizations according to the WHO classification (2016) and prevalence of associated Epstein–Barr virus infection in Nigeria population
Source: Infect Agent Cancer. 2021 May 24;16:36. doi: 10.1186/s13027-021-00378-z (PMC8142647; doi:10.1186/s13027-021-00378-z)
Supplement: Supplementary file 4 — Additional file 4. [file 13027_2021_378_MOESM4_ESM.docx]

**Supplementary Table 4:** Discordant diagnosis for Burkitt Lymphoma (n=3)

|  | Age | Gender | Biopsy site | Previous diagnosis | Revised diagnosis |
| --- | --- | --- | --- | --- | --- |
| 1 | 65 | M | Cervical lymph node | NHL | BL |
| 2 | 39 | M | Mesenteric lymph node | DLBCL, NOS | BL |
| 3 | 10 | M | Cervical lymph node | NHL | BL |
