# Supplementary material for: Distinct pattern of lymphoid neoplasms characterizations according to the WHO classification (2016) and prevalence of associated Epstein–Barr virus infection in Nigeria population
Source: Infect Agent Cancer. 2021 May 24;16:36. doi: 10.1186/s13027-021-00378-z (PMC8142647; doi:10.1186/s13027-021-00378-z)
Supplement: Supplementary file 5 — Additional file 5. [file 13027_2021_378_MOESM5_ESM.docx]

**Supplementary Table 5:** Remaining entities SMZL, ENMZL, FL, AITL, LPL, L-LBL (n=7)

|  | Age | Gender | Biopsy site | Previous diagnosis | Revised diagnosis |
| --- | --- | --- | --- | --- | --- |
| 1 | 68 | M | Cervical lymph node | NHL | FL |
| 2 | 39 | M | Spleen | CLL | SMZL |
| 3 | 71 | F | Spleen | CLL | SMZL |
| 4 | 48 | M | Conjunctiva | NK T-cell lymphoma | ENMZL |
| 5 | 28 | M | Cervical lymph node | DLBCL | AITL |
| 6 | 2 | M | Cervical lymph node | Reactive | B-LBL |
| 7 | 37 | M | Scalp | Plasmacytoma | LPL |
