# Supplementary material for: Distinct pattern of lymphoid neoplasms characterizations according to the WHO classification (2016) and prevalence of associated Epstein–Barr virus infection in Nigeria population
Source: Infect Agent Cancer. 2021 May 24;16:36. doi: 10.1186/s13027-021-00378-z (PMC8142647; doi:10.1186/s13027-021-00378-z)
Supplement: Supplementary file 6 — Additional file 6. [file 13027_2021_378_MOESM6_ESM.docx]

**Supplementary Table 6:** Cases reclassified as Non-Lymphoid Neoplasms.

|  | Age | Gender | Biopsy site | Previous diagnosis | Revised diagnosis |
| --- | --- | --- | --- | --- | --- |
| 1 | 17 | M | Caecal mass | NHL | Rosai Dorfman Disease |
| 2 | 17 | M | Cervical lymph node | NHL | Rosai Dorfman Disease |
| 3 | 46 | M | Cervical lymph node | LPL | Castelman Disease |
| 4 | 43 | M | Axillary lymph node | FL | Castelman Disease |
| 5 | 50 | M | Inguinal mass | NHL | Sarcoma |
| 6 | 40 | M | Abdominal-Inguinal mass | NHL | Sarcoma |
| 7 | 35 | F | Axillary lymph node | NHL | Sarcoma |
| 8 | 39 | M | Jejunum Intraabdominal mass | NHL | Sarcoma |
| 9 | 39 | M | Cervical lymph node | Reactive | Metastases of carcinoma |
| 10 | 12 | F | Axillary lymph node | Reactive | Metastases of carcinoma |
| 11 | 38 | F | Soft tissue | NHL | Metastases of carcinoma |
| 12 | 46 | M | Jejunum Intra-abdominal mass | NHL | Metastases of carcinoma |
| 13 | 38 | M | Left cervical lymph node | NHL | Metastases of carcinoma |
| 14 | 39 | M | Cervical lymph node | DLBCL | Metastases of carcinoma |
